# The 2025 ISCB Accomplishments by a Senior Scientist Award—Dr Amos Bairoch

**DOI:** 10.1093/bioinformatics/btaf262

**Published:** 2025-07-15

**Authors:** Mallory L Wiper

**Affiliations:** The International Society for Computational Biology, Leesburg, Virginia, United States

**Figure:**
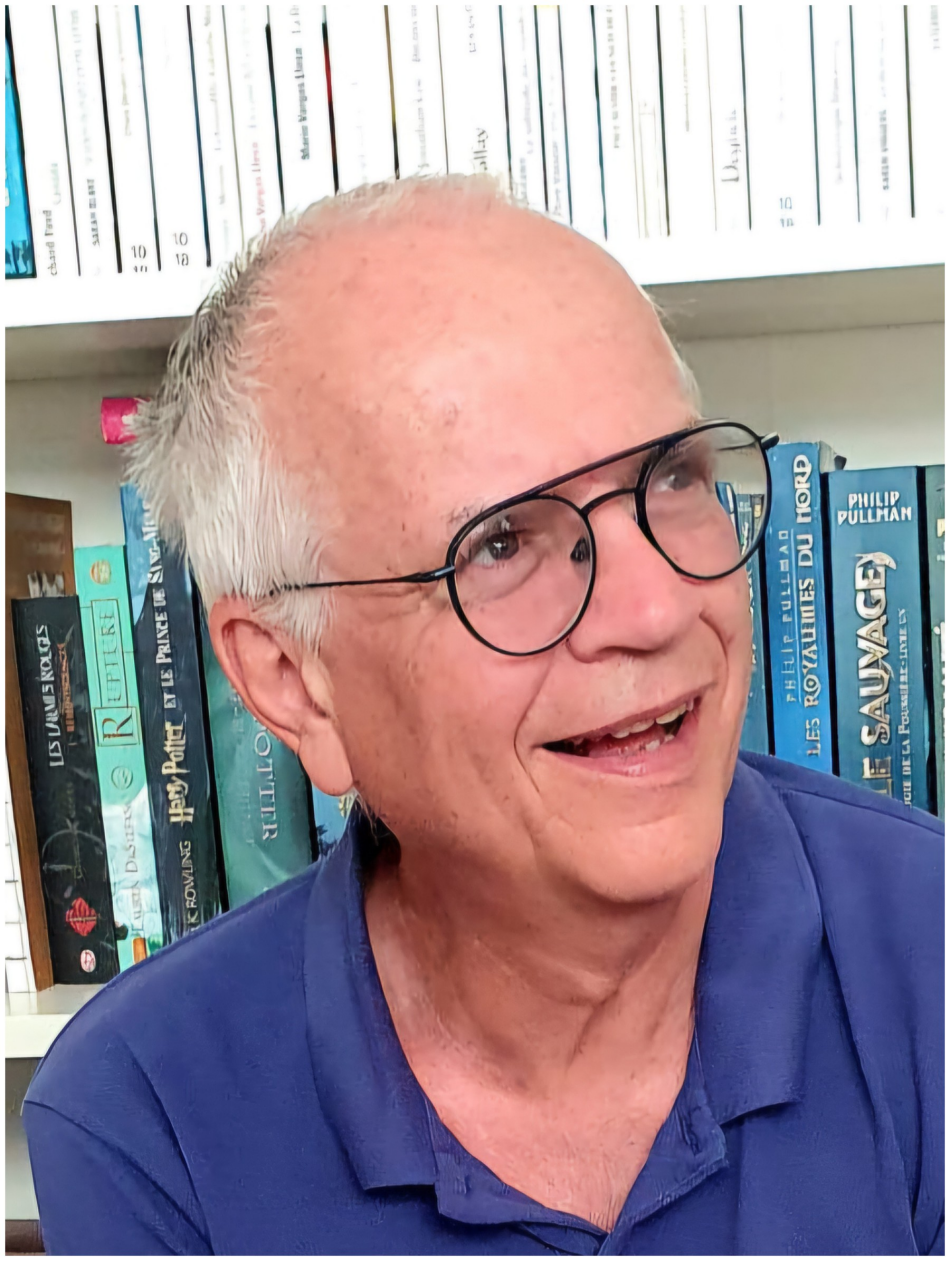


This year marks the 33rd annual conference on Intelligent Systems for Molecular Biology (ISMB) and the 24th European Conference on Computational Biology (ECCB). At this year’s meeting, being held in Liverpool, UK, the International Society for Computational Biology (ISCB) is proud to present the Accomplishments by a Senior Scientist Award to Dr Amos Bairoch in recognition of his leadership and innovation in the development of bioinformatics resources and infrastructure.

## A passion for discovery

While Dr Bairoch has been responsible for truly transformative advancements in bioinformatics, his interest in science started in childhood when his father gave him a book on astronomy. Bairoch recalls being fascinated by the contents of the book, sparking a deep interest in space and space exploration. Around age 10, he began collecting articles about the Apollo missions, following the space program with keen interest, and by age 12, Bairoch wanted to become an astronaut. That dream lasted until high school where he discovered that, at the time, a career as an astronaut required a military pilot background—something he wasn’t keen on pursuing.

Nevertheless, space and space exploration still fascinated Bairoch, so in university, he turned his attention to exobiology. The topic was gaining traction in the United States through figures like Carl Sagan, but it had not yet been established as a formal field of study in Switzerland or elsewhere in Europe. Not to be deterred from his passion for space exploration, Bairoch shifted his focus once again to biochemistry and mathematics, believing the two subjects to be an essential foundation for studying the chemical basis of life. It was his study of biochemistry converging with an interest in computers that ultimately brought Bairoch to the field of computational biology and bioinformatics.

## Beginnings in bioinformatics

Bairoch’s first steps on the path of computational biology began with a TRS-80 that his father, an economic historian, bought to run statistical analyses. Bairoch started writing programs to help his father conduct these analyses, which allowed him to experiment and explore the capabilities of computer programming. Around this time, he was reading papers from the labs of Rodger Staden and Richard Roberts, both of whom were developing tools for DNA and protein sequence analysis. These tools, however, were built for mainframe computers—the kind only available at major research institutions. But Bairoch saw another possibility: bringing sequence analysis to personal computers, making this type of analysis much more accessible.

By the end of his undergraduate studies, he brought the idea of sequence analysis on personal computers to Dr Robin Offord, a protein chemist interested in protein modification. Offord was intrigued by Bairoch’s idea and agreed to supervise him for his master’s thesis and later his PhD, though Offord recognized that he couldn’t fully assess the computational aspects of the work for the PhD project. Thus, he encouraged Bairoch to find a co-advisor with expertise in computational biology which led to Bairoch reaching out to Dr Jean-Michel Claverie at the Pasteur Institute in Paris. Claverie agreed to join the project as a co-advisor and every few months, Bairoch would travel to Paris to discuss his progress.

Though their early meetings were focused on his PhD work and next steps for the project, their discussions broadened beyond the PhD itself, especially as Bairoch’s focus shifted toward developing what would become Swiss-Prot.

Although Swiss-Prot wasn’t part of the original PhD project plan, it quickly became the centerpiece of his work, ultimately delaying the completion of his degree. Nevertheless, his advisors remained fully supportive, with Offord encouraging Bairoch to incorporate Swiss-Prot into the final thesis.

Without the staunch support of both advisors, Bairoch’s pioneering contributions to bioinformatics might never have taken shape.

## Support, mentorship, and scientific growth

There have been several influential people Bairoch has had the pleasure of working with, but when asked about the *most* influential mentors, he credited three key people with helping shape his academic research and bioinformatics career.

Notably was Dr Robin Offord who was one of the first people to support Bairoch’s idea of bringing sequence analysis to individual labs. Beyond the direct support from Offord, Bairoch later came to learn that Offord had sheltered him from harsh comments from other faculty regarding his PhD project at the University of Geneva. Most of the faculty felt there was no place for computers in life sciences, voicing concern that making a whole project surrounding this was a waste of time that could be better spent on research that wasn’t just “playing around with a computer.” Offord pushed back against that narrative, advocating for the value of computational approaches in biology. He even enlisted colleagues in the UK—where computational biology was gaining more traction—to help make the case. For Bairoch, the greatest lesson he learned from Offord was a model of mentorship that taught him that a professor’s role is not only to conduct research, but to help students achieve their goals. Without that early advocacy, Bairoch might never have found his way into bioinformatics.

A second key mentor in Bairoch’s career was his PhD co-advisor, Dr Jean-Michel Claverie, who provided intellectual guidance, reviewing Bairoch’s research and helping shape his approach to computational biology. Claverie not only provided bioinformatics insights that Offord was not fully able to provide, keeping Bairoch moving in the right direction with his PhD work, but he also provided camaraderie and meaningful discussions.

Finally, Bairoch named Doug Brutlag as a key mentor in his bioinformatics career. Brutlag, the creator of IntelliGenetics, a company developing software to analyze DNA and proteins, recognized the potential of Bairoch’s software (PC/Gene) and was instrumental in arranging its commercial distribution, the royalties of which allowed Bairoch to hire a research team to help him with the development of Swiss-Prot. Brutlag introduced him to the concept of technology transfer and provided an understanding of the commercialization of bioinformatics tools.

Bairoch’s own mentoring style was shaped by these key players in his career as well as by the unique circumstances of his work in bioinformatics. As Swiss-Prot expanded, his most significant mentoring impact has been through training biocurators, scientists responsible for annotating and maintaining high-quality biological data. In the absence of formal or standardized training programs in the field, Bairoch trained biocurators using an apprenticeship-style approach, where new curators learned through hands-on annotation work and received regular feedback and guidance.

With this approach to training new biocurators, Bairoch said that his mentoring style is similar to Offord’s. Namely, doing all he could to grant young researchers maximum creative freedom, while encouraging senior scientists, himself included, to step back and empower the next generation.

## From researcher to research leader

Bairoch’s early research career was highly independent, but as Swiss-Prot and the team around him grew, he had to transition from being a working scientist to being a research leader. He could no longer just be a colleague or collaborator—he had become a manager. With no clear roadmap for this transformation, he welcomed advice from scientists and management professionals and, over time, Bairoch learned that leading a large-scale research initiative required hierarchical structures, project management, and administrative oversight.

Recognizing that bioinformatics required such specialized support, Bairoch helped establish the Swiss Institute of Bioinformatics (SIB), an institutional framework designed to support the long-term stability of bioinformatics research in Switzerland.

The establishment of SIB meant that Bairoch’s role became less about individual research and more about ensuring the infrastructure and the team were successful, so he brought in Lydie Bougueleret to oversee administrative functions such as hiring and grant writing—tasks that had become too overwhelming to manage alongside scientific work. Through this transition, Bairoch learned to delegate leadership responsibilities and focus on building a system in which others could thrive.

## Unexpected directions and current fascinations

When asked what the most unexpected findings have been in his research, Bairoch had quite the unique answer! He said that there were no specific *findings*, but that what had intrigued him was the evolution of biological discoveries and how those discoveries impacted what software needed to be and do in order to keep up. For instance, Swiss-Prot started as small project and became the world’s leading protein sequence database used in biology and medicine. The management of its rapid growth required unexpected adaptations to make the product more widely available.

Another unexpected direction of his work was the accidental creation of Cellosaurus. While working on neXtProt, a database for human proteins, it came to Bairoch’s attention that there was no central database for cell lines, so he began a small side project to create such a resource, and this grew into Cellosaurus, a widely used resource for cell line information—and another example of how research and its supporting software continues to evolve.

This ongoing evolution—not just of databases, but of entire scientific fields—has remained a source of fascination for him.

## Reflections on winning the ISCB Accomplishments by a Senior Scientist Award

Bairoch said he was honored to be the recipient of the Accomplishments by a Senior Scientist Award. While he good-naturedly joked that a “young investigator” award would have been preferable—since senior scientist sounds like he’s at the end of his career!—he recognizes that this award is a reflection of his lifelong dedication to computational biology and bioinformatics and is truly grateful for the role he’s been able to play in shaping the field.

